# Retraction: Chen, Y.; Tao, J.; Wang, J.; Chen, X.; Xie, J.; Xiong, J.; Yang, K. The Novel Sensor Network Structure for Classification Processing Based on the Machine Learning Method of the ACGAN. *Sensors* 2019, *19*, 3145

**DOI:** 10.3390/s20020476

**Published:** 2020-01-15

**Authors:** By Sensors Editorial Office

**Affiliations:** MDPI, St. Alban-Anlage 66, 4052 Basel, Switzerland; sensors@mdpi.com

It has been brought to our attention that the majority of the text, figures, structures, and references in the title paper [1] is copied from a previously published paper written in Chinese [2]. The paper [1] will therefore be marked as retracted.

MDPI is a member of the Committee on Publication Ethics and takes seriously its responsibility to publish only high-quality research. We regret that this was not discovered during the review process and offer our apologies to the readers of *Sensors*.
